# Low on-treatment blood pressure and cardiovascular events in patients without elevated risk: a nationwide cohort study

**DOI:** 10.1038/s41440-024-01593-y

**Published:** 2024-02-14

**Authors:** Yuichiro Mori, Atsushi Mizuno, Shingo Fukuma

**Affiliations:** 1 Department of Human Health Sciences, Graduate School of Medicine, Kyoto University, Kyoto, Japan; 2 Department of Cardiovascular Medicine, St. Luke’s International Hospital, Tokyo, Japan

**Keywords:** Blood pressure, Cohort study, Coronary artery disease, Myocardial infarction, Stroke

## Abstract

Insufficient blood pressure control among patients with hypertension without elevated risk is a global concern, suggesting the need for treatment optimization. However, the potential harm of excessive blood pressure lowering among these patients is understudied. This study addressed this evidence gap by using nationally representative public health insurer database covering 30 million working-age population. Patients who were continuously using antihypertensive drugs with 10-year cardiovascular risk <10% were identified. They were categorized by on-treatment systolic and diastolic blood pressures. The primary outcome was a composite of myocardial infarction, stroke, heart failure hospitalization, and peripheral artery disease. Of 920,533 participants (mean age, 57.3 years; female, 48.3%; mean follow-up, 2.75 years), the adjusted hazard ratios for systolic blood pressure of <110, 110–119, 120–129 (reference), 130–139, 140–149, 150–159, and ≥160 mmHg were 1.05 (95% confidence interval: 0.99–1.12), 0.97 (0.93–1.02), 1 (reference), 1.05 (1.01–1.09), 1.15 (1.11–1.20), 1.30 (1.23–1.37), and 1.76 (1.66–1.86), respectively; and for diastolic blood pressure of <60, 60–69, 70–79 (reference), 80–89, 90–99, and ≥100 mmHg were 1.25 (1.14–1.38), 0.99 (0.95–1.04), 1 (reference), 1.00 (0.96–1.03), 1.13 (1.09–1.18), and 1.66 (1.58–1.76), respectively. Among low-risk patients with hypertension, diastolic blood pressure <60 mmHg was associated with increased cardiovascular events, while systolic blood pressure <110 mmHg was not. Compared to previous investigations in high-risk patients, the potential harm of excessive blood pressure lowering was less pronounced in low-risk patients with hypertension.

**Figure:**
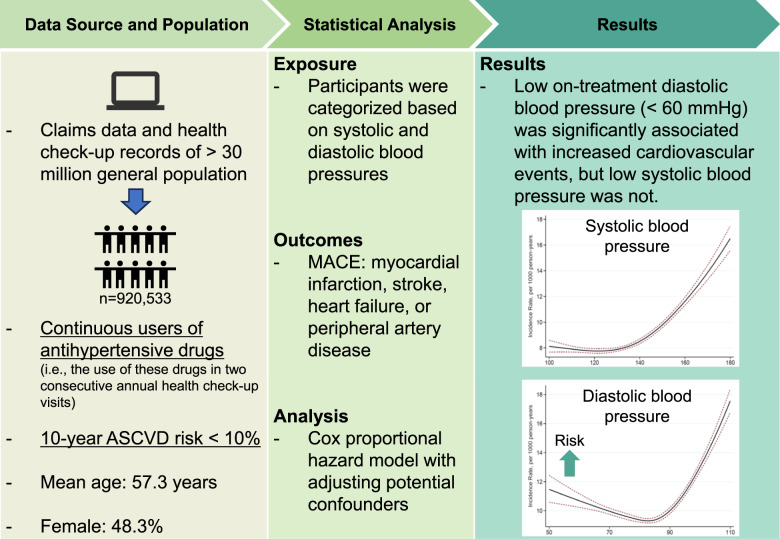
The association between low on-treatment blood pressure and cardiovascular events has been understudied in low-risk patients with hypertension. In our study with nationally representative working-age adults from general population with hypertension without elevated risk, increased risk of cardiovascular events was observed in diastolic blood pressure of <60 mmHg, but not in systolic blood pressure of <110 mmHg. Those results contrasted with previous investigations in high-risk patients where the risk of low on-treatment blood pressure was more pronounced.

The association between low on-treatment blood pressure and cardiovascular events has been understudied in low-risk patients with hypertension. In our study with nationally representative working-age adults from general population with hypertension without elevated risk, increased risk of cardiovascular events was observed in diastolic blood pressure of <60 mmHg, but not in systolic blood pressure of <110 mmHg. Those results contrasted with previous investigations in high-risk patients where the risk of low on-treatment blood pressure was more pronounced.

## Abstract (web summary)

The association between low on-treatment blood pressure and cardiovascular events has been understudied in low-risk patients with hypertension. In our study with nationally representative working-age adults from general population with hypertension without elevated risk, increased risk of cardiovascular events was observed in diastolic blood pressure of <60 mmHg, but not in systolic blood pressure of <110 mmHg. Those results contrasted with previous investigations in high-risk patients where the risk of low on-treatment blood pressure was more pronounced.

## Introduction

Hypertension is a leading cause of cardiovascular disease (CVD). In 2019, hypertension accounted for >20% of all deaths worldwide [1], and about half of the patients treated for hypertension still had on-treatment blood pressure (BP) > 140/90 mmHg [2]. To mitigate the global burden of hypertension, intensifying treatments for patients with low cardiovascular risk is vital because a majority of patients with hypertension have 10-year atherosclerotic CVD (ASCVD) risks <10% [3, 4], and such low-risk patients are reportedly more likely to be uncontrolled than patients with higher risk [5]. However, the potential harm of excessive BP lowering is not well investigated in that patient population. Filling this evidence gap is imperative for physicians to optimize hypertension treatment for patients with low cardiovascular risk.

To the best of our knowledge, evidence for the potential harm of low on-treatment BP has been long discussed, but the discussion was limited to high-risk patients [6, 7]. Recently, two large observational studies in high-risk patients reported an increased risk of cardiovascular outcomes in low on-treatment systolic BP (SBP) of <120 mmHg and diastolic BP (DBP) of <70 mmHg [8, 9]. Based on these reports, the 2018 European Society of Cardiology and the European Society of Hypertension guidelines suggested maintaining SBP above 120 mmHg for patients with hypertension in general [10].

Thus, the potential harm of low on-treatment BP in low-risk patients is yet to be studied, presumably due to challenges in the required number of patients and length of follow-up for adequate statistical analysis [11]. To address this knowledge gap, we used nationally representative data from the working-age general population in Japan to investigate the relationship between on-treatment BP and cardiovascular outcomes among low-risk patients with hypertension.

Point of view

- Clinical relevance:
  In patients with a 10-year atherosclerotic cardiovascular risk below 10%, maintaining a low on-treatment blood pressure would be safe provided that the diastolic blood pressure remained above 60 mmHg.
- Future direction:
  Clinical trials comparing blood pressure control strategies with multiple control targets in patients with low cardiovascular risk
- Consideration for the Asian population:
  Considering that the harm of low blood pressure is often attributed to reduced coronary artery flow, the Asian population, which predominantly experiences stroke as a major cardiovascular event, might exhibit a better tolerance to lower blood pressure levels than other populations.

## Methods

The present study is a longitudinal analysis of administrative and medical record data. The study was reported in accordance with the Strengthening the Reporting of Observational Studies in Epidemiology guidelines [12] (Supplementary Table 1).

### Data source

We used health insurance claims data and annual health check-up records offered by the largest health insurer in Japan (Japan Health Insurance Association), which provides public health insurance for employed workers and their families. The insurer covers more than 30 million individuals (32% of the country’s entire population) [13]. Data were extracted from insurance claims records and annual health check-up results, including laboratory data. Practice and death records were linked with a disease code that can be translated into the International Classification of Diseases, 10th revision (ICD-10).

The details of Japan’s government-led annual health check-up program have been previously described [14–16]. Every government-certified public health insurer in Japan, including the Japan Health Insurance Association, is required to provide general health check-ups to members aged 40–74 years once in a fiscal year (April–March). According to the data provider, the attendance rate of the health check-up program in 2020 was 52.3% among ~18 million eligible adults [17]. The check-ups include BP measurements, laboratory testing, and self-reporting of medical and behavioral status. The BP was measured in a casual setting (i.e., in a clinic or hospital with the attendance of healthcare providers; measurements were mostly automated). Reporting the mean of two BP measurements conducted in a check-up was the standard protocol. Self-reporting questionnaires were manually checked by healthcare professionals (in many cases, trained nurses) at the site of health check-ups.

### Population

This study targeted patients with a low CVD risk who were treated with antihypertensive drugs. The study population was retrospectively identified from working-age (40–74 years) insured members who attended health check-ups in two consecutive fiscal years for the first time from April 1, 2015 to March 31, 2021. The latter of the two check-ups was defined as the baseline, in which on-treatment BP and other baseline characteristics were identified. The inclusion criteria were (i) The use of antihypertensive drugs in both two visits (i.e., had been using antihypertensive drugs in a certain duration); (ii) No prior CVD; and (iii) 10-year ASCVD risk [4] <10%. We adopted a 10-year ASCVD risk threshold of 10% to define low CVD risk in accordance with the latest guidelines [10, 11]. Participants were excluded if they met at least one of the following criteria: (i) missing data in variables used in the main analysis; (ii) recorded DBP higher than SBP (impossible values), (iii) history of cancer or end-stage renal disease; (iv) receiving medications for diabetes mellitus; (v) A1c (HbA1c) ≥ 6.5%; and (vi) fasting blood glucose (FBG) ≥ 126 mg/dL. Patients with diabetes were excluded because optimal blood pressure for that population has already been extensively investigated [18, 19], and the American Diabetes Association provided a guideline recommendation [20]. Medical histories were confirmed with interviews at health check-ups except for cancer (no interviews were conducted), which was ascertained with claims data during the observation period prior to the baseline check-ups (ICD-10: C00-97, D00-09, and D37-48).

### Exposures and predictors

The study cohort was categorized based on their SBP (<110, 110–119, 120–129 [reference], 130–139, 140–149, 150–159, and ≥160) and DBP (<60, 60–69, 70–79 [reference], 80–89, 90–99, and ≥100) measured at the baseline health check-up (i.e., the latter of two consecutive heath check-up with the use of antihypertensive drugs). Referential standards were determined based on current guideline recommendations and suggestions for low-risk patients with hypertension [10, 11]. Other predictor variables adjusted in the statistical analysis included age, sex, low-density lipoprotein cholesterol (LDL-C), triglyceride, high-density lipoprotein cholesterol (HDL-C), body mass index (BMI), receiving medications for dyslipidemia (yes/no), having prediabetes (yes if: HbA1c of ≥5.7 and <6.5; or FBG of ≥101 and <126 [20]) and smoking status (yes if: more than 100 cigarettes lifetime, smoking duration longer than 6 months, and the last smoke within a month). HbA1c and FBG were summarized to a binary variable of prediabetes (yes/no) because the health check-up program measure either of them only.

### Outcomes

The primary outcomes were acute myocardial infarction (AMI), stroke, heart failure (HF), and peripheral artery disease (PAD). Events were identified as hospitalization or death records in the insurance claims data linked with relevant disease codes based on the ICD-10 (AMI: I20–25, Stroke: I60–69, HF: I50, PAD: I70). The secondary outcomes were each component of the composite outcome. All patients were followed until March 31, 2021, when the outcome occurred or when insurance coverage was lost (treated as lost to follow-up).

### Statistical analysis

A Cox proportional hazards model was used to evaluate the association between the baseline on-treatment BP and cardiovascular outcomes. The hazard ratio (HR) of each BP category compared to the reference was calculated by adjusting for predictor variables. As there should be a strong correlation between SBP and DBP, analyses were performed independently for SBP and DBP to avoid collinearity. Restricted cubic spline models with four knots were applied for continuous variables included in the model (age, LDL-C, HDL-C, triglyceride, and BMI). We then conducted the same analysis for the secondary outcomes. Two additional analyses were also conducted: (1) the overall trend of association between BP and the primary composite outcome was visualized with a restricted cubic spline smoothing technique in which BP was treated as a continuous variable, and (2) the main analysis was repeated with cross-classified systolic and blood pressure categories, in which categories that included less than 500 patients were excluded to secure a sufficient number of events for adequate statistical analysis. Statistical analyses were performed using Stata version 17.0 (Stata Corp., College Station, TX, USA).

### Sensitivity and subgroup analyses

We conducted two sensitivity analyses. First, to account for the changes in on-treatment BP over time, we handled BP records in health check-ups after the baseline as a time-varying exposure in the Cox proportional hazard model, adjusting the same baseline variables as the main analysis. Second, to account for misclassifications of BP categories due to a limited number of measurements, we reclassified BP categories by averaging the BP records in the two consecutive annual health check-ups that were part of the eligibility criteria. In addition, we conducted subgroup analyses based on age (40–49, 50–59, or ≥60 years), sex, BMI (<18.5, 18.5–24.9, or ≥25), dyslipidemia status (LDL-C ≥ 140 mg/dL or receiving medications for dyslipidemia), and smoking status.

### Ethical review of study

The Institutional Review Board of Kyoto University approved this study and waived the requirement of informed consent owing to the use of de-identified data (R2913). All methods were conducted in accordance with the Declaration of Helsinki.

## Results

**Fig. 1:**
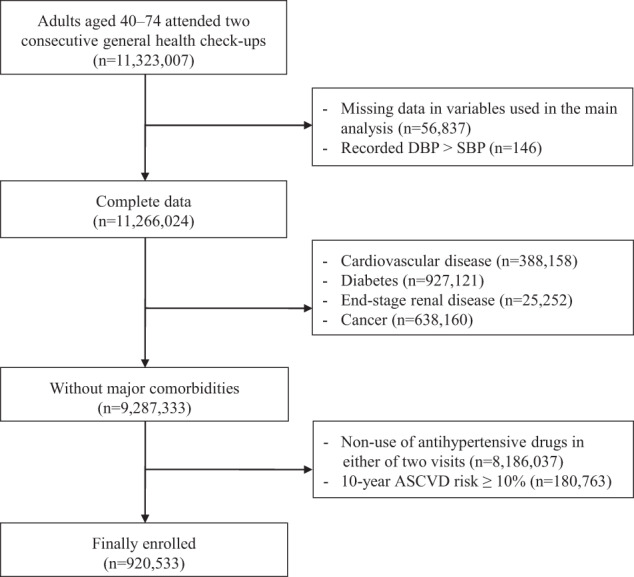
Patient selection. The variables used in the main analysis included age, sex, low-density lipoprotein cholesterol, triglyceride, high-density lipoprotein cholesterol, body mass index, medications for dyslipidemia (yes/no), and smoking status (yes/no). Individuals with missing values in at least one of those variables or blood pressure were excluded. ASCVD atherosclerotic cardiovascular disease, DBP diastolic blood pressure, SBP systolic blood pressure

From 11,323,007 working-age (40–74) adults, 920,533 low-risk patients treated with antihypertensive drugs in two consecutive health check-ups were included (Fig. 1). Only 0.5% (56,837/11,323,007) were excluded because of missing or impossible data (i.e., DBP > SBP). The mean (standard deviation: SD) age was 57.3 (6.9) years, 48.3% (*n* = 445,053/920,533) were female, the mean BMI was 24.9 (3.9) kg/m^2^, and the mean 10-year ASCVD risk was 3.6 (2.7)% (Table 1). The intervals between the two consecutive health check-ups were mostly around 365 days (median [interquartile range]: 365 days [357–378]). The mean on-treatment SBP and DBP were 131.7 (15.4) mmHg and 81.8 (10.8) mmHg, respectively (Supplementary Table 2). Patients with lower BP categories tended to be older, have lower BMI, and have higher smoking rates (Tables 1 and 2). The proportion of patients who were controlled below 140/90 mmHg and below 130/80 mmHg, the guideline-recommended level, was 65.8% (605,946/920,533) and 30.9% (284,214/920,533), respectively (Supplementary Table 3).

**Table 1:** Baseline characteristics according to systolic blood pressure categories

Systolic blood pressure, mmHg	<110	120–129	130–139	140–149	150–159	150–159	≥160	Overall
	*n* = 53,909	*n* = 131,101	*n* = 245,858	*n* = 239,226	*n* = 139,968	*n* = 68,227	*n* = 42,244	*n* = 920,533
Age, mean (SD), y	58.1 (7.1)	57.6 (7.1)	57.5 (7.0)	57.3 (6.9)	57.0 (6.8)	56.6 (6.6)	55.8 (6.5)	57.3 (6.9)
Female, No. (%)	27,261 (50.6)	64,286 (49.0)	119,250 (48.5)	114,543 (47.9)	66,175 (47.3)	32,523 (47.7)	21,015 (49.7)	445,053 (48.3)
Body mass index, mean (SD), kg/m^2^	23.8 (3.5)	24.3 (3.6)	24.7 (3.7)	25.1 (3.9)	25.3 (4.1)	25.5 (4.3)	25.8 (4.7)	24.9 (3.9)
LDL-C, mean (SD), mg/dL	116.9 (28.0)	118.7 (27.5)	119.8 (27.8)	120.7 (28.1)	121.9 (28.6)	123.0 (29.4)	125.1 (30.7)	120.5 (28.3)
Triglyceride, mean (SD), mg/dL	118.2 (78.2)	121.9 (78.5)	125.2 (80.7)	127.5 (83.3)	129.0 (84.0)	129.2 (84.5)	130.1 (85.7)	126.0 (82.0)
HDL-C, mean (SD), mg/dL	61.5 (16.6)	61.5 (16.3)	61.8 (16.3)	62.2 (16.4)	62.6 (16.8)	63.2 (17.0)	64.0 (17.8)	62.2 (16.6)
Prediabetes^a^, No. (%)	20,803 (38.6)	52,668 (40.2)	101,924 (41.5)	102,907 (43.0)	63,012 (45.0)	31,367 (46.0)	19,947 (47.2)	392,628 (42.7)
Medications for dyslipidaemia, No. (%)	15,946 (29.6)	38,544 (29.4)	69,638 (28.3)	65,163 (27.2)	35,437 (25.3)	16,454 (24.1)	9027 (21.4)	250,209 (27.2)
Smoking, No. (%)	16,463 (30.5)	36,159 (27.6)	62,054 (25.2)	56,432 (23.6)	31,720 (22.7)	14,808 (21.7)	9323 (22.1)	226,959 (24.7)
10-year ASCVD risk^b^, mean (SD), %	2.8 (2.3)	3.1 (2.5)	3.5 (2.6)	3.8 (2.7)	4.0 (2.8)	4.2 (2.8)	4.4 (2.8)	3.6 (2.7)

*ASCVD* atherosclerotic cardiovascular disease, *HDL-C* high-density lipoprotein cholesterol, *LDL-C* low-density lipoprotein cholesterol

^a^HbA1c ≥ 5.7% and < 6.5% or FBG ≥ 101 mg/dL and < 126 mg/dL (those with HbA1c ≥ 6.5% or FBG ≥ 126 mg/dL were excluded in the study)

^b^Based on the American College of Cardiology and the American Heart Association pooled cohort equation

**Table 2:** Baseline characteristics according to diastolic blood pressure categories

Diastolic blood pressure, mmHg	<60	60–69	70–79	80–89	90–99	≥100
	*n* = 13,826	*n* = 91,896	*n* = 273,530	*n* = 337,283	*n* = 155,680	*n* = 48,318
Age, mean (SD), y	60.1 (7.3)	59.4 (6.9)	58.3 (6.8)	57.0 (6.8)	55.6 (6.7)	53.7 (6.6)
Female, No. (%)	8451 (61.1)	53,752 (58.5)	147,099 (53.8)	156,321 (46.3)	62,313 (40.0)	17,117 (35.4)
Body mass index, mean (SD), kg/m^2^	23.7 (3.7)	24.1 (3.7)	24.5 (3.8)	25.0 (3.9)	25.5 (4.1)	26.1 (4.5)
LDL-C, mean (SD), mg/dL	116.1 (28.4)	117.8 (27.7)	119.5 (27.6)	120.7 (28.1)	122.5 (29.0)	124.9 (30.6)
Triglyceride, mean (SD), mg/dL	113.1 (72.3)	116.2 (72.4)	121.0 (76.6)	127.4 (83.2)	134.2 (89.0)	140.8 (93.6)
HDL-C, mean (SD), mg/dL	62.7 (17.0)	62.6 (16.4)	62.4 (16.4)	62.2 (16.6)	61.7 (16.7)	61.4 (17.2)
Prediabetes^a^, No. (%)	5665 (41.0)	38,013 (41.4)	114,391 (41.8)	143,860 (42.7)	69,065 (44.4)	21,634 (44.8)
Medications for dyslipidaemia, No. (%)	4592 (33.2)	29,837 (32.5)	82,131 (30.0)	89,133 (26.4)	35,414 (22.7)	9102 (18.8)
Smoking, No. (%)	3740 (27.1)	23,698 (25.8)	67,150 (24.5)	81,306 (24.1)	37,970 (24.4)	13,095 (27.1)
10-year ASCVD risk^b^, mean (SD), %	3.6 (2.7)	3.6 (2.7)	3.7 (2.7)	3.6 (2.7)	3.7 (2.7)	3.6 (2.7)

*ASCVD* atherosclerotic cardiovascular disease, *HDL-C* high-density lipoprotein cholesterol, *LDL-C* low-density lipoprotein cholesterol

^a^HbA1c ≥ 5.7% and < 6.5% or FBG ≥ 101 mg/dL and < 126 mg/dL (those with HbA1c ≥ 6.5% or FBG ≥ 126 mg/dL were excluded in the study)

^b^Based on the American College of Cardiology and the American Heart Association pooled cohort equation

The mean follow-up duration was 2.75 years. Overall, 22,833 primary outcomes were observed (9.03 per 1000 patient-years, 95% confidence interval [CI]: 8.91–9.15). Of these, only 825 deaths occurred (0.32 [0.30–0.34] per 1000 patient-years). The incidences for components of the composite outcome (AMI, stroke, HF, and PAD) were 3.73 [3.66–3.81], 3.96 [3.88–4.04], 2.72 [2.66–2.79], and 0.77 [0.74–0.81] per 1000 patient-years, respectively (Supplementary Table 4).

**Fig. 2:**
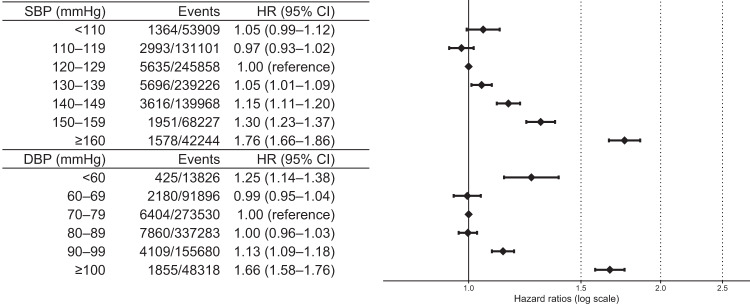
Adjusted hazard ratios (95% CI) of the primary outcomes according to systolic and diastolic blood pressure. The analysis was adjusted for age, sex, low-density lipoprotein cholesterol, triglyceride, high-density lipoprotein cholesterol, body mass index, medications for dyslipidemia (yes/no), smoking status (yes/no), and 10-year ASCVD risk calculated from general health check-up results. Restricted cubic spline models with four knots were applied to the continuous variables included in the model. ASCVD atherosclerotic cardiovascular disease, CI confidence interval DBP diastolic blood pressure, SBP systolic blood pressure, HR hazard ratio

**Fig. 3:**
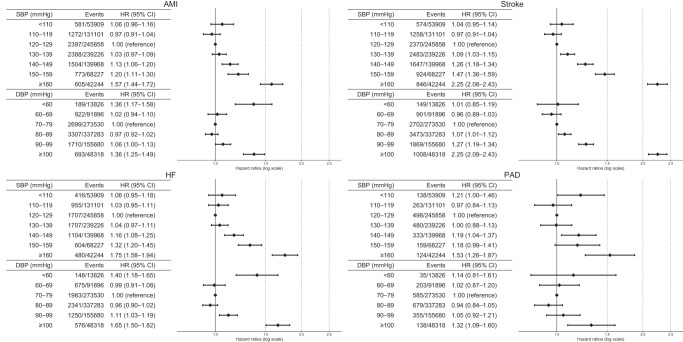
Adjusted hazard ratios (95% CI) of the secondary outcomes according to systolic and diastolic blood pressure. Adjusted for age, sex, low-density lipoprotein cholesterol, triglyceride, high-density lipoprotein cholesterol, body mass index, medications for dyslipidemia (yes/no), smoking status (yes/no), and 10-year ASCVD risk calculated from general health check-up results. Restricted cubic spline models with four knots were applied to the continuous variables included in the model. ASCVD atherosclerotic cardiovascular disease, CI confidence interval, DBP diastolic blood pressure, HF heart failure, HR hazard ratio, MI myocardial infarction, PAD peripheral arterial disease, SBP systolic blood pressure

After adjusting for potential confounders, the HRs for the SBP of <110 mmHg, 110–119 mmHg, 120–129 mmHg (reference), 130–139 mmHg, 140–149 mmHg, 150–159 mmHg, and ≥160 mmHg were 1.05 (95% confidence interval: 0.99–1.12), 0.97 (0.93–1.02), 1 (reference), 1.05 (1.01–1.09), 1.15 (1.11–1.20), 1.30 (1.23–1.37), and 1.76 (1.66–1.86), respectively; and for DBP of <60 mmHg, 60–69 mmHg, 70–79 mmHg (reference), 80–89 mmHg, 90–99 mmHg, and ≥100 mmHg were 1.25 (1.14–1.38), 0.99 (0.95–1.04), 1 (reference), 1.00 (0.96–1.03), 1.13 (1.09–1.18), and 1.66 (1.58–1.76), respectively (Fig. 2). In all BP categories, events occurred almost steadily throughout the follow-up period (Supplementary Fig. 1). Regarding secondary outcomes, DBP < 60 mmHg was associated with increased risks of AMI and HF, whereas the association between SBP < 110 and cardiovascular outcomes was significant in all components. When comparing the association of low BP and four secondary outcomes, the risk of low DBP seemed to be more emphasized in AMI and HF than in stroke. This difference was not obvious in SBP (Fig. 3).

**Fig. 4:**
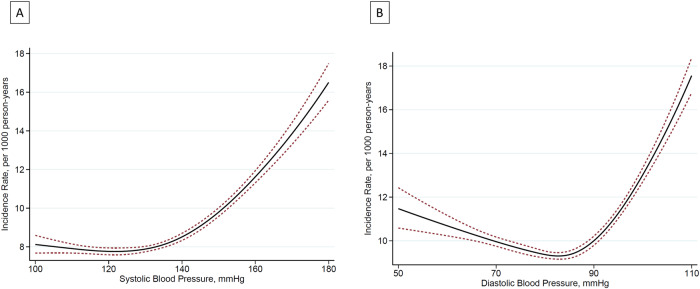
Restricted cubic splines of the primary outcome with systolic (**A**) and diastolic (**B**) blood pressure. The associations between the baseline systolic/diastolic blood pressure and the primary composite outcome were visualized using a Poisson regression model with a restricted cubic spline technique. The model was adjusted for the same covariates as the main analysis, and the incidence rates were estimated using the model and mean covariate values for all participants. DBP diastolic blood pressure, SBP systolic blood pressure

In the additional analysis that handled BP as a continuous function, the lowest incidence was observed in those with SBP of ~120–130 mmHg and DBP of ~80 mmHg with an increased incidence with low DBP (Fig. 4). Another additional analysis with cross-classified SBP and DBP categories also demonstrated a consistently increased incidence in DBP < 60 mmHg, while this increase related to low DBP was mitigated in those with low SBP compared to those with normal SBP range (HR [95% CI]: 1.18 [1.03–1.35] with SBP < 110 mmHg; 1.12 [0.90–1.39] with SBP 110–119 mmHg; 1.54 [1.19–1.99] with SBP was 120–129 mmHg; and 1.45 [0.99–2.14] with SBP 130–139 mmHg) (Supplementary Fig. 2).

In all sensitivity and subgroup analyses, the overall trend—the increasing trend in the primary outcome in low DBP but not significantly in low SBP—was almost consistent. For the sensitivity analyses, DBP < 60 mmHg was consistently associated with increased risk even when BP categories were determined by averaging the measurements in two consecutive years or when BP measurements during the follow-up were considered as time-varying exposures (Supplementary Figs. 3 and 4). In the subgroup analysis, male sex, higher age, absence of dyslipidemia, and current smoking exhibited stronger associations between low DBP and increased risk. On the other hand, BMI seemed to have little or no interaction. The comprehensive results of the subgroup analyses are displayed in Supplementary Tables 5 and 6.

## Discussion

In this nationwide cohort of low-risk patients who were prevalent users of antihypertensive drugs, on-treatment DBP < 60 mmHg was associated with increased cardiovascular events, whereas on-treatment DBP of 60–69 mmHg and SBP < 110 mmHg were not. About 30% of the cohort was controlled at a guideline-recommended target of <130/80 mmHg. As for the current state of potential harm associated with excess BP lowering, only 1.5% of the cohort were at elevated risk related to low on-treatment BP (i.e., DBP < 60 mmHg). This analysis included patients who have a 10-year ASCVD risk <10% and were free from major risk-modifying complications such as cancer.

Our findings advance the current state of knowledge regarding the potential harm of low on-treatment BP in low-risk patients with hypertension. In a previous large observational study for patients with elevated CVD risk, the increase in incidence was observed in DBP < 70 mmHg and SBP < 120 mmHg [8]. Our results suggest that low BP could be more tolerable in low-risk patients than in high-risk patients. The strength of the present study is the use of annual health check-up results and claims data from nationwide samples. This large cohort enabled us to assess potential risks among low-risk patients. In addition, utilizing general health check-up results made it possible to identify low-risk patients who would be difficult to be ascertained only with claims data.

The association between low on-treatment BP and increased CVD risk, named the J-curve phenomenon, has been debated for decades [6–9, 21–24] and was mainly observed in the risk of heart-related events (e.g., AMI and HF) [8]. The underlying mechanism of this discrepancy has been proposed to be decreased blood flow into the coronary arteries during the diastolic phase. Indeed, our results also showed the association between low BP and increased incidence clearer in AMI and HF than in stroke. Given these contexts, the extent of BP control intensification might be modified based on what kind of CVD events (i.e., heart-related or stroke) are primarily concerned in the patient. In addition, patients with low DBP should be cautious about the signs of heart-related symptoms.

Previously, concerns about reverse causality have always been proposed regarding the J-curve phenomenon [6, 7]. The present study is also not completely free from the same concern that patients with already declining health status were likely to be classified into low BP categories. For example, there was a pronounced increase in CVD events associated with low DBP among those without dyslipidemia compared to those with dyslipidemia. This result may be due to those with declining health status at baseline, which would introduce both low cholesterol level and low DBP. To deal with this point, we limited participants to prevalent users (i.e., continuously treated with antihypertensive drugs for two consecutive annual health checkups) and excluded patients with prior cancer diagnoses. In addition, the incidence rate was almost constant throughout the observation period, even in the lowest BP category. Since severely ill patients would experience events shortly after the start of follow-up, this steady survival curve suggests that the impact of reverse causality due to severely ill patients at the baseline would be limited at least in the overall study cohort even if they could not be fully excluded.

Of note, our results observed the increased risk related to low on-treatment BP only in DBP but not significantly in SBP, while prior studies in high-risk patients reported the increased risk in SBP < 120 mmHg [8, 9]. This discrepancy between our results and previous research might be explained by the long-discussed hypothesis that the J-curve phenomenon is primarily due to decreased diastolic blood flow in coronary arteries [6, 7, 21]. As an elevated CVD risk is associated with an increase in pulse pressure [25], low-risk patients would be capable of tolerating low SBP in terms of maintained DBP. Indeed, when compared to the previous observational study in patients with elevated risk [8], our cohort had more patients with SBP < 120 mmHg (about 20% vs. 12%), while the proportion of patients with DBP < 60 mmHg was almost the same (around 1%). The analysis with cross-classified SBP and DBP categories also suggested no significant incidence increase associated with low SBP if DBP was adequately maintained (e.g., 60–80 mmHg). These findings indicate that the prior reports about the J-curve phenomenon in on-treatment SBP could have owed to the decline in DBP.

In our cohort, about 65% of low-risk patients treated with antihypertensive drugs have their on-treatment BP less than 140/90 mmHg. This percentage is better than the global average, which is estimated at ~50% [2]. On the other hand, our cohort showed only ~1.5% of patients presented with significantly elevated risk due to excessive BP reduction (i.e., DBP < 60 mmHg). This small proportion would support the potential safety of hypertension treatment for such low-risk patients. Given these findings, there would be room for physicians in many countries to intensify hypertension treatments among low-risk patients if appropriate risk stratifications were conducted and caution was paid for maintaining DBP above a safety threshold, which might be around 60–70 mmHg.

The present study has some limitations. First, despite the extensive eligibility assessment to exclude patients with elevated risk of CVD events, there would remain concerns about unadjusted confounding and reverse causality. Second, there remain possibilities of misclassification in exposure because of the limited number of BP measurements. Third, the white-coat effect can affect casual BP measurement in the presence of healthcare providers [26]. Conversely, it has a strength in its application in usual clinical settings [27]. Fourth, though our database does not include information on race/ethnicity, most of the sturdy cohort is assumed to be Asian. Last, our analysis did not account for the antihypertensive drug classes. Further investigations would focus on the external validity of our results among participants with various social backgrounds and on the influence of treatment regimens on the results.

### Perspective of Asia

The importance of stroke prevention has been emphasized more in Asian populations than in other regions [28]. Our findings indicate that the potential harm of low on-treatment BP is less apparent in stroke outcomes than in AMI or HF. Given that the majority of patients with hypertension in Asia are undiagnosed or uncontrolled [2], these insights would guide physicians in Asia to safely optimize blood pressure control in patients with low cardiovascular risk.

## Conclusion

Among patients with low cardiovascular risk, DBP < 60 mmHg was associated with an increased incidence of cardiovascular events, while SBP < 110 mmHg was not. Compared to previous investigations in high-risk patients, the potential harm of excessive blood pressure lowering was less pronounced in low-risk patients with hypertension.

### Supplementary information

Supplementary Materials
