# Supplementary material for: Low on-treatment blood pressure and cardiovascular events in patients without elevated risk: a nationwide cohort study
Source: Hypertens Res. 2024 Feb 14;47(6):1546–54. doi: 10.1038/s41440-024-01593-y (PMC11150151; doi:10.1038/s41440-024-01593-y)
Supplement: Supplementary file 1 — Supplementary Materials [file 41440_2024_1593_MOESM1_ESM.docx]

**Supplementary Materials**

**Low On-Treatment Blood Pressure and Cardiovascular Events in Patients without Elevated Risk: A Nationwide Cohort Study**

**Table of contents**

**Supplementary Tables**

Supplementary Table 1 The STROBE checklist

Supplementary Table 2: Distribution of systolic and diastolic blood pressure among participants

Supplementary Table 3: The proportions of participants by systolic and diastolic blood pressure categories

Supplementary Table 4: Follow-up duration and incidence rate for the primary outcome, each component of the composite outcome, and cardiovascular death.

Supplementary Table 5: Subgroup analysis by systolic blood pressure categories

Supplementary Table 6: Subgroup analysis by diastolic blood pressure categories

**Supplementary Figures**

Supplementary Figure 1: Kaplan-Meier survival curve for the primary outcome by systolic (left) and diastolic (right) blood pressure categories

Supplementary Figure 2: Association between on-treatment blood pressure and cardiovascular events by cross-classified systolic and diastolic blood pressure categories

Supplementary Figure 3: Sensitivity analysis with reclassifying blood pressure with averages of measurements at two consecutive health check-ups

Supplementary Figure 4: Sensitivity analysis with blood pressure after inclusion as time-varying exposure

**Supplementary** **Table 1 The STROBE checklist**

**STROBE 2007 (v4) Statement—Checklist of items that should be included in reports of *cohort studies***

**Title: Low On-Treatment Blood Pressure and Cardiovascular Events in Patients without Elevated Risk: A Nationwide Cohort Study**

**Authors: Yuichiro Mori, MD, MPH, Atsushi Mizuno, MD, MBA, PhD, Shingo Fukuma, MD, PhD**

| **Section/Topic** | Item # | Recommendation | Reported on page # |
| --- | --- | --- | --- |
| **Title and abstract** | 1 | (*a*) Indicate the study’s design with a commonly used term in the title or the abstract | Title |
|  |  | (*b*) Provide in the abstract an informative and balanced summary of what was done and what was found | 1-2 |
| Introduction | | |  |
| Background/rationale | 2 | Explain the scientific background and rationale for the investigation being reported | 4 |
| Objectives | 3 | State specific objectives, including any prespecified hypotheses | 4 |
| Methods | | |  |
| Study design | 4 | Present key elements of study design early in the paper | 5 |
| Setting | 5 | Describe the setting, locations, and relevant dates, including periods of recruitment, exposure, follow-up, and data collection | 5 |
| Participants | 6 | (*a*) Give the eligibility criteria, and the sources and methods of selection of participants. Describe methods of follow-up | 5-6 |
|  |  | (*b*) For matched studies, give matching criteria and number of exposed and unexposed | NA |
| Variables | 7 | Clearly define all outcomes, exposures, predictors, potential confounders, and effect modifiers. Give diagnostic criteria, if applicable | 6 |
| Data sources/ measurement | 8* | For each variable of interest, give sources of data and details of methods of assessment (measurement). Describe comparability of assessment methods if there is more than one group | 5 |
| Bias | 9 | Describe any efforts to address potential sources of bias | 7 |
| Study size | 10 | Explain how the study size was arrived at | NA |
| Quantitative variables | 11 | Explain how quantitative variables were handled in the analyses. If applicable, describe which groupings were chosen and why | 6-7 |
| Statistical methods | 12 | (*a*) Describe all statistical methods, including those used to control for confounding | 7-8 |
|  |  | (*b*) Describe any methods used to examine subgroups and interactions | 8, |
|  |  | (*c*) Explain how missing data were addressed | 6 |
|  |  | (*d*) If applicable, explain how loss to follow-up was addressed | 7 |
|  |  | (*e*) Describe any sensitivity analyses | 7-8 |
| Results | | |  |
| Participants | 13* | (a) Report numbers of individuals at each stage of study—eg numbers potentially eligible, examined for eligibility, confirmed eligible, included in the study, completing follow-up, and analysed | 8 |
|  |  | (b) Give reasons for non-participation at each stage | 8 |
|  |  | (c) Consider use of a flow diagram | Figure 1 |
| Descriptive data | 14* | (a) Give characteristics of study participants (eg demographic, clinical, social) and information on exposures and potential confounders | 8-9 |
|  |  | (b) Indicate number of participants with missing data for each variable of interest | 8 |
|  |  | (c) Summarise follow-up time (eg, average and total amount) | 8-9 |
| Outcome data | 15* | Report numbers of outcome events or summary measures over time | 8-9 |
| Main results | 16 | (*a*) Give unadjusted estimates and, if applicable, confounder-adjusted estimates and their precision (eg, 95% confidence interval). Make clear which confounders were adjusted for and why they were included | 9 |
|  |  | (*b*) Report category boundaries when continuous variables were categorized | 9 |
|  |  | (*c*) If relevant, consider translating estimates of relative risk into absolute risk for a meaningful time period | NA |
| Other analyses | 17 | Report other analyses done—eg analyses of subgroups and interactions, and sensitivity analyses | 9-10 |
| Discussion |  |  |  |
| Key results | 18 | Summarise key results with reference to study objectives | 10 |
| **Limitations** |  |  |  |
| Interpretation | 20 | Give a cautious overall interpretation of results considering objectives, limitations, multiplicity of analyses, results from similar studies, and other relevant evidence | 12 |
| Generalisability | 21 | Discuss the generalisability (external validity) of the study results | 11-12 |
| Other information |  |  |  |
| Funding | 22 | Give the source of funding and the role of the funders for the present study and, if applicable, for the original study on which the present article is based | 14 |

**Supplementary** **Table 2:** **Distribution of systolic and diastolic blood pressure among participants**

| Percentiles | SBP |  |  | Summary Statistics | |
| --- | --- | --- | --- | --- | --- |
| Min | 58 |  |  | Mean | 131.7 |
| 1% | 99 |  |  | SD | 15.4 |
| 5% | 108 |  |  |  |  |
| 10% | 113 |  |  |  |  |
| 25% | 122 |  |  |  |  |
|  |  |  |  |  |  |
| 50% | 130 |  |  |  |  |
|  |  |  |  |  |  |
| 75% | 140 |  |  |  |  |
| 90% | 151 |  |  |  |  |
| 95% | 159 |  |  |  |  |
| 99% | 174 |  |  |  |  |
| Max | 277 |  |  |  |  |
|  |  |  |  |  |  |
| Percentiles | DBP |  |  | Summary Statistics | |
| Min | 19 |  |  | Mean | 81.8 |
| 1% | 58 |  |  | SD | 10.8 |
| 5% | 64 |  |  |  |  |
| 10% | 69 |  |  |  |  |
| 25% | 75 |  |  |  |  |
|  |  |  |  |  |  |
| 50% | 81.5 |  |  |  |  |
|  |  |  |  |  |  |
| 75% | 88 |  |  |  |  |
| 90% | 95 |  |  |  |  |
| 95% | 100 |  |  |  |  |
| 99% | 109 |  |  |  |  |
| Max | 172 |  |  |  |  |

DBP, diastolic blood pressure; SBP, systolic blood pressure; SD, standard deviation

**Supplementary Table 3: The proportions of participants by systolic and diastolic blood pressure categories**

| n (%) |  |  | |  | | DBP, mmHg | |  | |  | |  | |  |
| --- | --- | --- | --- | --- | --- | --- | --- | --- | --- | --- | --- | --- | --- | --- |
|  |  | < 60 | 60**–**69 | | 70–79 | | 80–89 | | 90-99 | | ≥ 100 | | Overall | |
|  | < 110 | 7,834 (0.9%) | 26,326 (2.9%) | | 17,691 (1.9%) | | 2,004 (0.2%) | | 50 (0.0%) | | 4 (0.0%) | | 53,909 (5.9%) | |
|  | 110-119 | 3,079 (0.3%) | 30,808 (3.4%) | | 69,776 (7.6%) | | 26,084 (2.8%) | | 1,334 (0.1%) | | 20 (0.0%) | | 131,101 (14.2%) | |
| SBP, mmHg | 120–129 | 1,745 (0.2%) | 22,565 (2.5%) | | 104,390 (11.3%) | | 102,807 (11.2%) | | 13,997 (1.5%) | | 354 (0.0%) | | 245,858 (26.7%) | |
|  | 130–139 | 784 (0.1%) | 8,847 (1.0%) | | 58,620 (6.4%) | | 122,586 (13.3%) | | 44,911 (4.9%) | | 3,478 (0.4%) | | 239,226 (26.0%) | |
|  | 140–149 | 258 (0.0%) | 2,408 (0.3%) | | 17,197 (1.9%) | | 57,740 (6.3%) | | 51,894 (5.6%) | | 10,471 (1.1%) | | 139,968 (15.2%) | |
|  | 150–159 | 84 (0.0%) | 700 (0.1%) | | 4,414 (0.5%) | | 19,470 (2.1%) | | 29,320 (3.2%) | | 14,239 (1.6%) | | 68,227 (7.4%) | |
|  | ≥ 160 | 42 (0.0%) | 242 (0.0%) | | 1,442 (0.2%) | | 6,592 (0.7%) | | 14,174 (1.5%) | | 19,752 (2.2%) | | 42,244 (4.6%) | |
|  | Overall | 13,826 (1.5%) | 91,896 (10.0%) | | 273,530 (29.7%) | | 337,283 (36.6%) | | 155,680 (16.9%) | | 48,318 (5.3%) | | 920,533 (100.0%) | |

DBP, diastolic blood pressure; SBP, systolic blood pressure

**Supplementary** **Table 4: Follow-up duration and incidence rate for the primary outcome, each component of the composite outcome, and cardiovascular death**.

|  | n | Mean follow-up duration, years | Events | Incidence rate per 1000 patient-year (95% CI) |
| --- | --- | --- | --- | --- |
| MACE | 920,533 | 2.75 | 22,833 | 9.03 (8.91–9.15) |
| Acute myocardial infarction | 920,533 | 2.77 | 9,520 | 3.73 (3.66–3.81) |
| Stroke | 920,533 | 2.77 | 10,102 | 3.96 (3.88–4.04) |
| Heart failure | 920,533 | 2.78 | 6,973 | 2.72 (2.66–2.79) |
| Peripheral arterial disease | 920,533 | 2.78 | 1,995 | 0.77 (0.74–0.81) |
| Cardiovascular death | 920,533 | 2.79 | 825 | 0.32 (0.30–0.34) |

MACE indicates the primary composite endpoint of acute myocardial infarction, stroke, heart failure, or peripheral arterial disease.

CI, confidence interval; MACE, major adverse cardiovascular event

**Supplementary** **Table 5: Subgroup analysis by systolic blood pressure categories**

|  |  |  | Systolic blood pressure, mmHg | | |  |  |  |  |
| --- | --- | --- | --- | --- | --- | --- | --- | --- | --- |
| Subgroup | n | < 110 | 110–129 | 120–129 | 130–139 | 140–149 | 150–159 | ≥ 160 | p-value |
| Female | 445,053 | 1.10 (1.00–1.21) | 0.98 (0.91–1.06) | 1 (ref.) | 1.06 (1.00–1.13) | 1.15 (1.08–1.24) | 1.23 (1.12–1.34) | 1.45 (1.32–1.60) | <0.001 |
| Male | 475,480 | 1.04 (0.96–1.12) | 0.97 (0.92–1.03) | 1 (ref.) | 1.04 (0.99–1.09) | 1.15 (1.09–1.21) | 1.33 (1.24–1.42) | 1.93 (1.80–2.07) |  |
| BMI < 20.0, kg/m^2^ | 68,989 | 1.05 (0.88–1.26) | 0.98 (0.84–1.14) | 1 (ref.) | 1.06 (0.92–1.22) | 1.31 (1.12–1.53) | 1.21 (0.99–1.49) | 1.79 (1.45–2.23) | 0.53 |
| 20.0 ≤ BMI < 25.0, kg/m2 | 444,416 | 1.04 (0.95–1.13) | 0.98 (0.92–1.05) | 1 (ref.) | 1.04 (0.98–1.09) | 1.13 (1.06–1.20) | 1.25 (1.15–1.35) | 1.62 (1.48–1.78) |  |
| BMI > 25.0, kg/m2 | 407,128 | 1.08 (0.98–1.19) | 0.96 (0.90–1.03) | 1 (ref.) | 1.06 (1.00–1.12) | 1.16 (1.09–1.23) | 1.35 (1.26–1.45) | 1.85 (1.71–2.00) |  |
| Age 40-49 years | 160,264 | 0.95 (0.79–1.14) | 0.93 (0.82–1.05) | 1 (ref.) | 1.09 (0.99–1.20) | 1.28 (1.15–1.43) | 1.47 (1.30–1.67) | 2.29 (2.02–2.59) | <0.001 |
| Age 50-59 years | 383,283 | 1.13 (1.03–1.24) | 0.94 (0.88–1.01) | 1 (ref.) | 1.03 (0.98–1.10) | 1.13 (1.06–1.20) | 1.33 (1.23–1.43) | 1.85 (1.71–2.00) |  |
| Age ≥ 60 years | 376,986 | 1.03 (0.94–1.12) | 1.01 (0.95–1.08) | 1 (ref.) | 1.05 (0.99–1.10) | 1.13 (1.06–1.21) | 1.18 (1.08–1.29) | 1.30 (1.17–1.45) |  |
| Non-dyslipidemia | 488,180 | 1.11 (1.03–1.20) | 1.00 (0.94–1.06) | 1 (ref.) | 1.09 (1.03–1.14) | 1.17 (1.11–1.24) | 1.34 (1.25–1.44) | 1.87 (1.73–2.02) | 0.004 |
| Dyslipidemia | 432,353 | 0.99 (0.91–1.09) | 0.95 (0.89–1.01) | 1 (ref.) | 1.00 (0.95–1.06) | 1.13 (1.07–1.20) | 1.25 (1.16–1.35) | 1.62 (1.49–1.77) |  |
| Non-smoker | 693,574 | 1.07 (0.99–1.15) | 0.99 (0.93–1.04) | 1 (ref.) | 1.04 (0.99–1.08) | 1.11 (1.05–1.17) | 1.23 (1.16–1.31) | 1.59 (1.49–1.71) | <0.001 |
| Smoker | 226,959 | 1.03 (0.93–1.13) | 0.95 (0.88–1.03) | 1 (ref.) | 1.08 (1.01–1.15) | 1.26 (1.17–1.36) | 1.45 (1.32–1.60) | 2.17 (1.97–2.40) |  |

Values were hazard ratios (95% confidence intervals).

P values were evaluated by the likelihood ratio test.

Dyslipidemia indicates LDL-C ≥ 140 mg/dL or receiving medications for dyslipidemia.

BMI, body mass index; ref., reference

**Supplementary Table 6: Subgroup analysis by diastolic blood pressure categories**

|  |  |  | Diastolic blood pressure, mmHg | | |  |  |  |
| --- | --- | --- | --- | --- | --- | --- | --- | --- |
| Subgroup | n | < 60 | 60**–**69 | 70–79 | 80–89 | 90-99 | ≥ 100 | p-value |
| Female | 445,053 | 1.18 (1.02–1.36) | 0.98 (0.91–1.06) | 1 (ref.) | 1.03 (0.98–1.09) | 1.20 (1.12–1.28) | 1.46 (1.31–1.63) | <0.001 |
| Male | 475,480 | 1.34 (1.17–1.53) | 1.01 (0.95–1.08) | 1 (ref.) | 0.98 (0.94–1.02) | 1.10 (1.04–1.15) | 1.71 (1.61–1.82) |  |
| BMI < 20.0, kg/m^2^ | 68,989 | 1.12 (0.85–1.48) | 0.82 (0.70–0.97) | 1 (ref.) | 0.99 (0.87–1.11) | 1.09 (0.93–1.27) | 1.79 (1.45–2.20) | 0.39 |
| 20.0 ≤ BMI < 25.0, kg/m2 | 444,416 | 1.36 (1.20–1.56) | 1.04 (0.97–1.11) | 1 (ref.) | 1.01 (0.96–1.06) | 1.15 (1.08–1.22) | 1.65 (1.51–1.80) |  |
| BMI > 25.0, kg/m2 | 407,128 | 1.13 (0.94–1.35) | 0.98 (0.91–1.06) | 1 (ref.) | 0.99 (0.94–1.04) | 1.12 (1.06–1.19) | 1.65 (1.54–1.78) |  |
| Age 40-49 years | 160,264 | 1.03 (0.71–1.51) | 0.84 (0.71–1.00) | 1 (ref.) | 1.02 (0.93–1.12) | 1.18 (1.06–1.31) | 2.01 (1.80–2.25) | <0.001 |
| Age 50-59 years | 383,283 | 1.30 (1.08–1.57) | 1.05 (0.96–1.14) | 1 (ref.) | 0.98 (0.93–1.03) | 1.16 (1.09–1.23) | 1.65 (1.53–1.78) |  |
| Age ≥ 60 years | 376,986 | 1.27 (1.12–1.43) | 0.99 (0.93–1.06) | 1 (ref.) | 1.01 (0.96–1.06) | 1.08 (1.01–1.15) | 1.30 (1.16–1.46) |  |
| Non-dyslipidemia | 488,180 | 1.43 (1.26–1.63) | 1.05 (0.98–1.12) | 1 (ref.) | 1.02 (0.98–1.07) | 1.17 (1.10–1.23) | 1.68 (1.56–1.80) | <0.001 |
| Dyslipidemia | 432,353 | 1.07 (0.92–1.25) | 0.94 (0.88–1.01) | 1 (ref.) | 0.97 (0.92–1.02) | 1.09 (1.03–1.16) | 1.65 (1.52–1.78) |  |
| Non-smoker | 693,574 | 1.22 (1.08–1.37) | 0.97 (0.91–1.03) | 1 (ref.) | 0.97 (0.93–1.01) | 1.10 (1.05–1.15) | 1.53 (1.43–1.64) | <0.001 |
| Smoker | 226,959 | 1.33 (1.12–1.57) | 1.05 (0.97–1.15) | 1 (ref.) | 1.05 (0.99–1.12) | 1.20 (1.12–1.29) | 1.95 (1.78–2.13) |  |

Values were hazard ratios (95% confidence intervals).

P values were evaluated by the likelihood ratio test.

Dyslipidemia indicates LDL-C ≥ 140 mg/dL or receiving medications for dyslipidemia.

BMI, body mass index; ref., reference

**Supplementary** **Figure 1: Kaplan-Meier survival curve for the primary outcome by systolic (left) and diastolic (right) blood pressure categories**


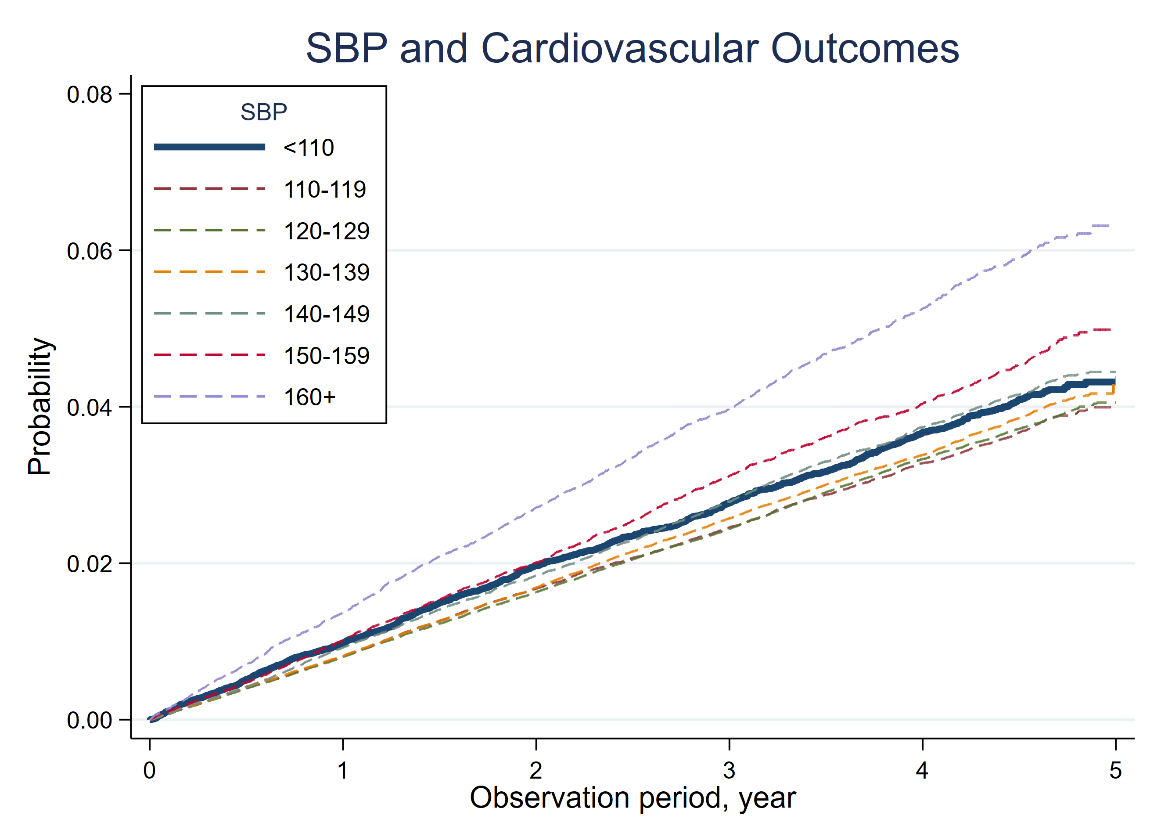


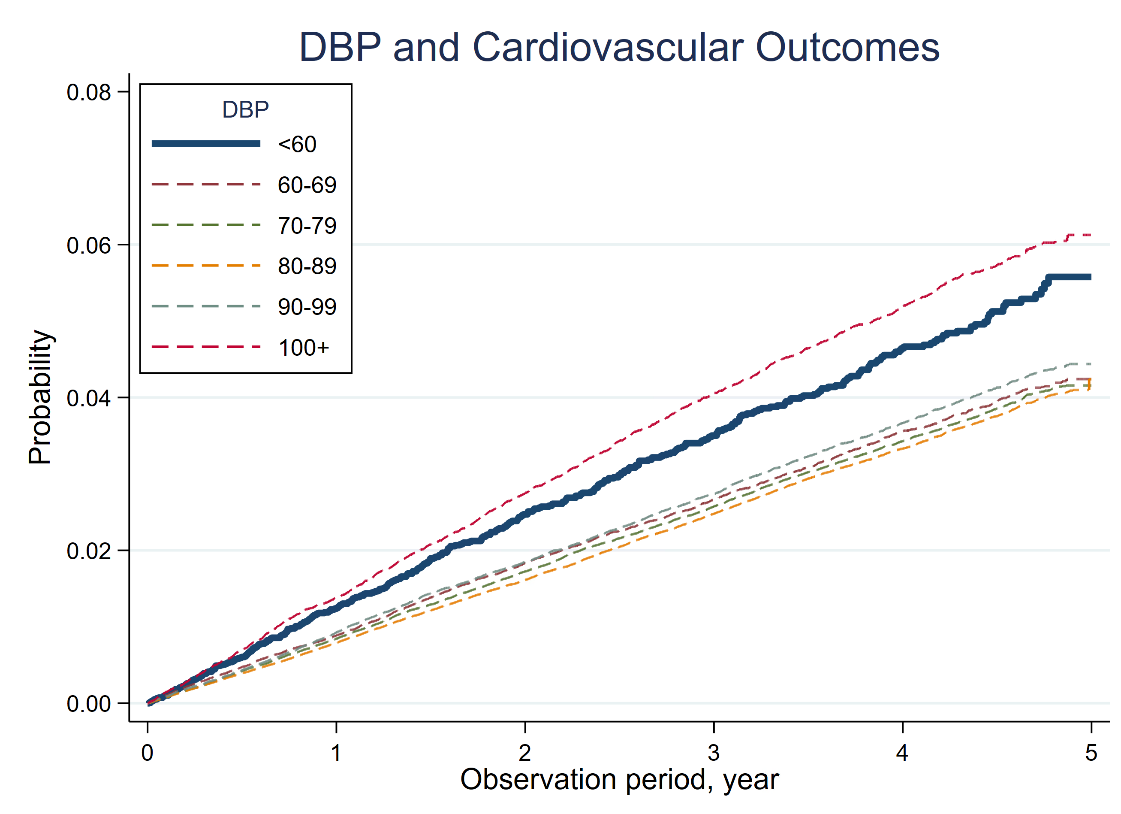


DBP, diastolic blood pressure; SBP, systolic blood pressure

**Supplementary Figure 2**: Association between on-treatment blood pressure and cardiovascular events by cross-classified systolic and diastolic blood pressure categories


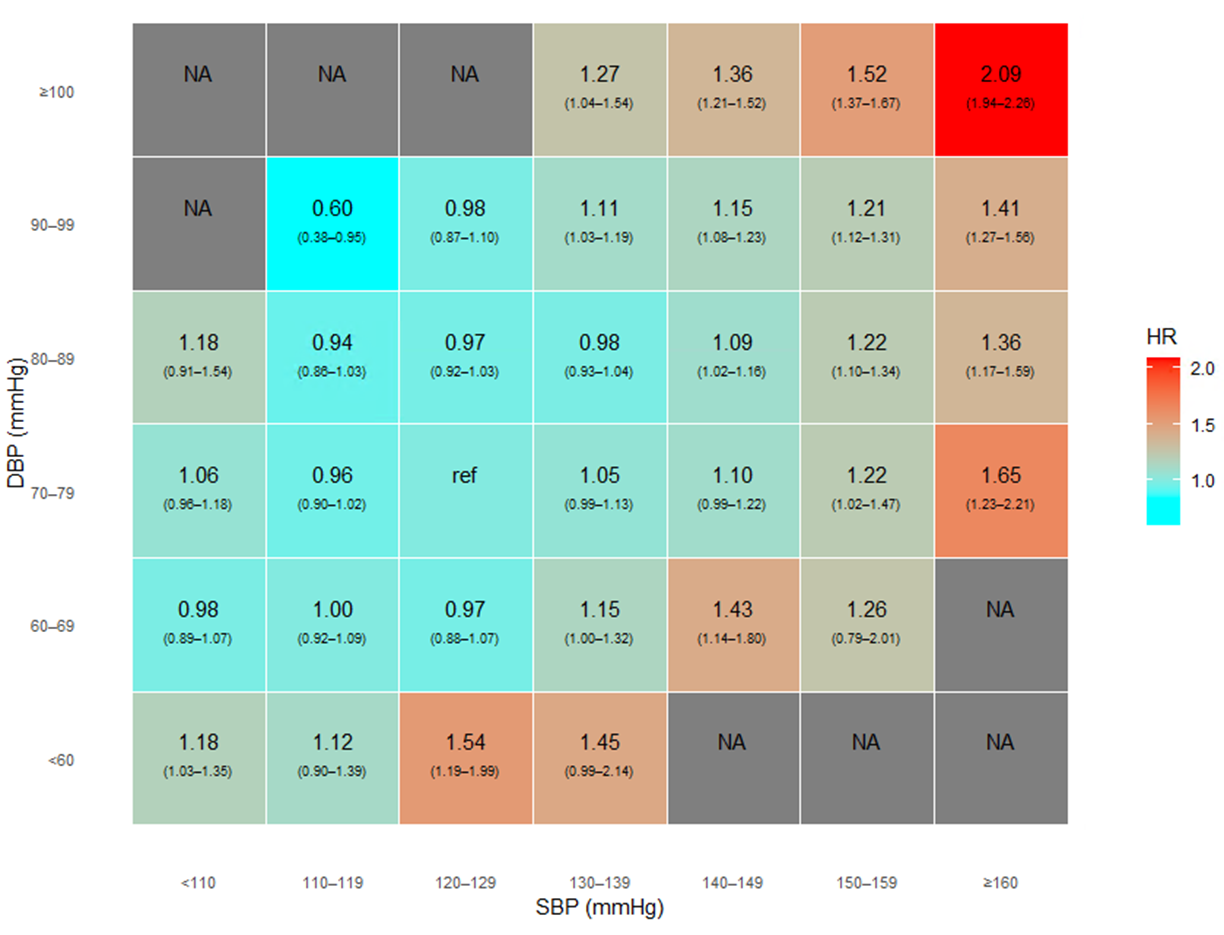


The HRs and 95% confidence intervals (parentheses) were calculated with a Cox proportional hazard model which included the same covariates and splines with the main analysis and a categorical variable of the cross-classified systolic and diastolic blood pressure categories. Categories that include less than 500 patients were excluded to secure enough number of events for adequate statistical analysis. Referential standard was set in line with the main analysis (SBP of 120–129 mmHg and DBP of 70–79 mmHg).

DBP, diastolic blood pressure; HR, hazard ratio; NA, not applicable; ref, referential standard; SBP, systolic blood pressure

**Supplementary** **Figure 3: Sensitivity analysis with reclassifying blood pressure with averages of measurements at two consecutive health check-ups**


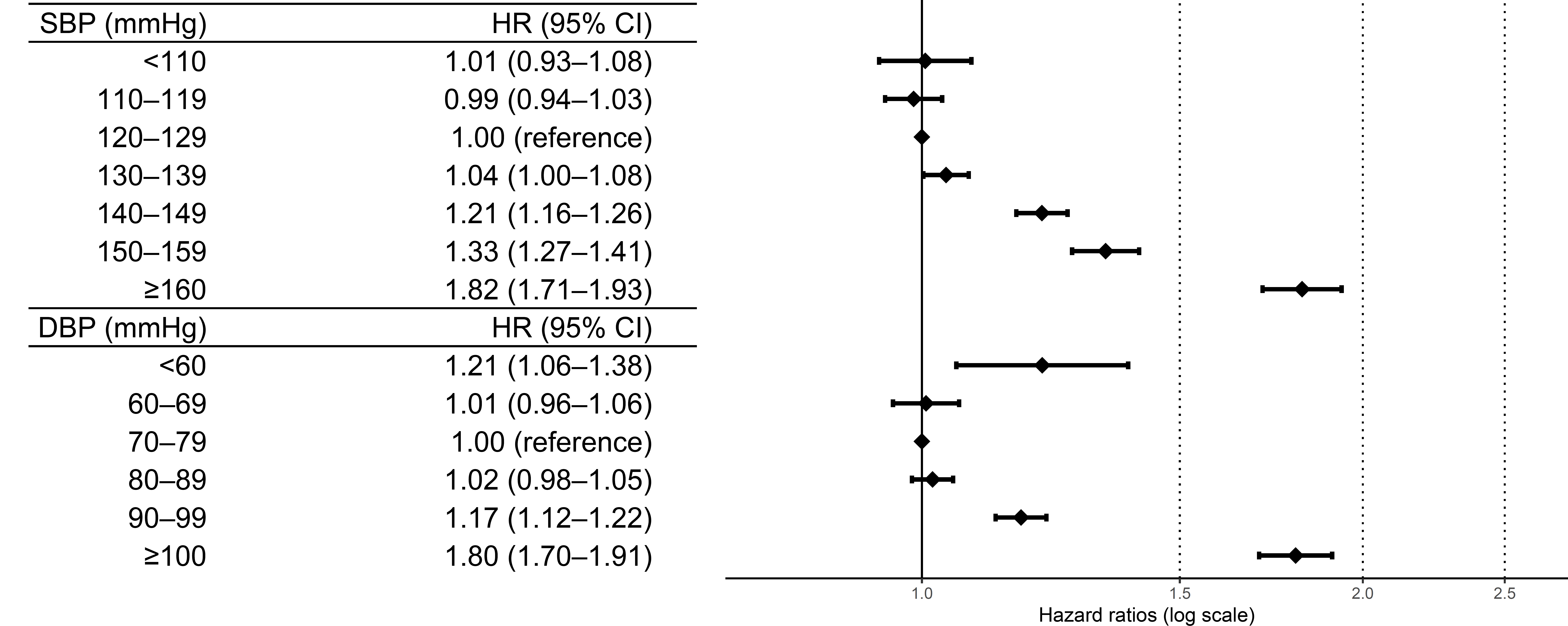


CI, confidence interval; DBP, diastolic blood pressure; HR, hazard ratio; SBP, systolic blood pressure

**Supplementary Figure 4: Sensitivity analysis with blood pressure after inclusion as time-varying exposures**


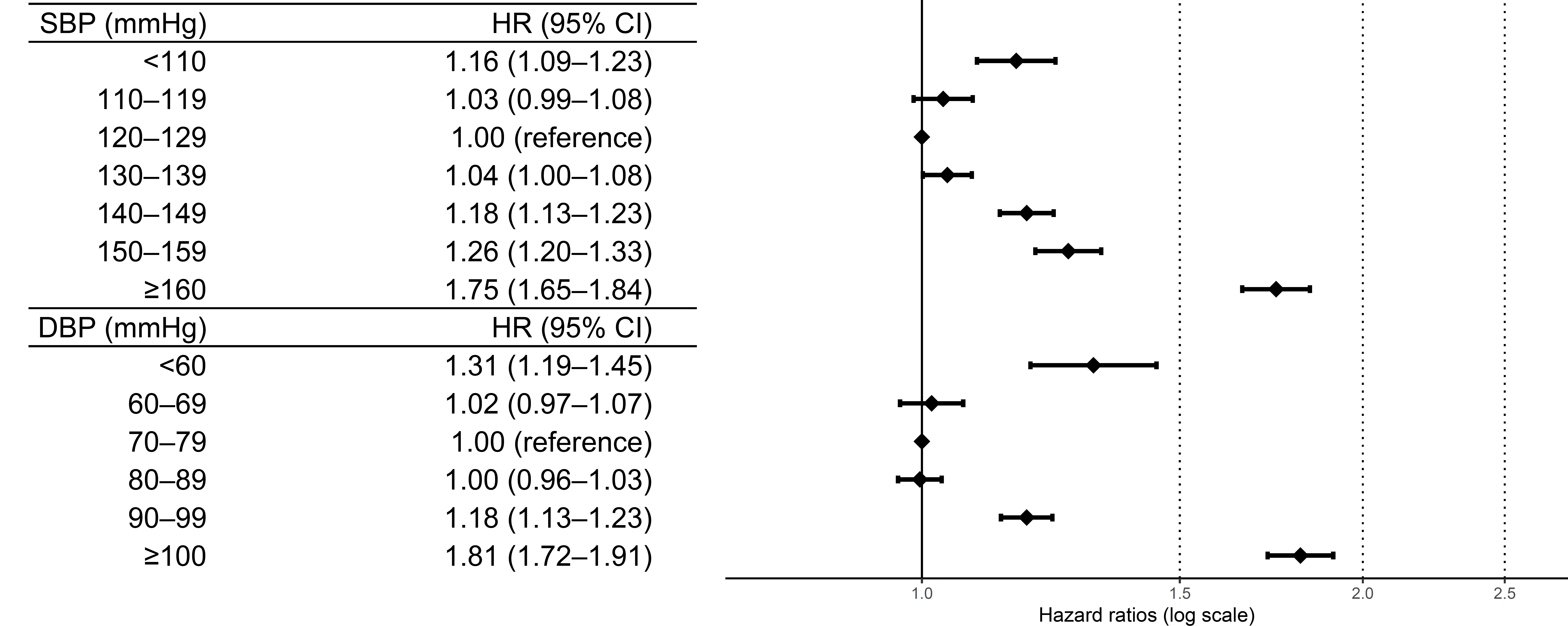


CI, confidence interval; DBP, diastolic blood pressure; HR, hazard ratio; SBP, systolic blood pressure
